# The Role of Chief Medical officers in making Public Health Overview Documents in Norwegian Municipalities. A qualitative Study

**DOI:** 10.1186/s12889-024-18608-5

**Published:** 2024-04-23

**Authors:** Dag-Helge Rønnevik, Betty J. Pettersen, Anders Grimsmo, Aslak Steinsbekk

**Affiliations:** 1 ISM, NTNU, Trondheim, Norway; 2 Municipality of Trondheim, Trondheim, Norway

**Keywords:** Chief Medical Officer, Public health, Community medicine, Epidemiology, Preventive medicine, General practice

## Abstract

**Aims:**

To investigate how Chief Medical Officers experience their role in the municipalities´ work with making the public health overview documents, demanded by the Norwegian Public Health Act from 2012.

**Methods:**

A qualitative study with semi-structured focus group interviews with 21 Chief Medical Officers from 20 different municipalities in Norway. The interviews were conducted in 2017. The data were analyzed thematically.

**Results:**

The Chief Medical Officers were mainly positive to participating in making public health overview documents. They took on roles as leaders of the work, medical advisors, data collectors towards local GPs and listening post to other sectors. Organizational factors like too small positions and a lack of tradition to involve the CMO in public health work were experienced as barriers to their involvement. The collaboration with the public health coordinators was said to be rewarding, and the intersectoral process involved employees from other sectors in a new way in public health. Although there were some positive experiences, several CMOs considered the use and impact of the public health overview document as limited.

**Conclusion:**

There was a large variation in the amount and the type of involvement the Chief Medical Officers had in making the public health overview documents in Norwegian municipalities. More research is needed to understand if this has any consequences for the quality of public health work in the municipalities and whether it is a sign of a changing role of the Chief Medical Officers.

## Background

The role of Chief Medical Officers (CMOs), also termed public health physicians, medical officer of health and district medical officer, has developed over time [1–3]. From 1984 it became mandatory for Norwegian municipalities to have one or more CMO as medical advisor [4].

Two thirds of the 356 Norwegian municipalities have only one CMO, and about half of all CMOs have combined positions as general practitioners (GPs), often with the CMO position as a small add on, typical 20% of a man-year [5]. The position as a CMO may cover public health, infection control, environmental health care, primary health care management, quality assurance and planning processes, often in a hybrid role as advisors on different managerial levels [6]. During the COVID-19 pandemic, CMOs in Norway as well as in other countries were heavily involved in infection control and vaccination programs and they were publicly exposed through communication with the general population, management, and politicians [7, 8].

The Public health Act (PHA) introduced in 2012 [9], stated that Norwegian municipalities must involve all sectors in promoting public health, and have a public health overview document. The overview document shall identify the public health challenges in the municipality, including assessment of causal factors and impact on population health. It should be designed to be used as a base for the municipality’s planning and policy development, also in accordance with the Planning and Building Act [10], representing a ‘Health in All Policies’ (HiAP) as proposed by the WHO [11, 12]. The medical expertise represented by the CMOs is stated in the PHA as a prerequisite for this work. One or more CMOs must be employed as medical advisors for the municipality to take care of community medical advice in the municipality’s public health work, including epidemiological analyses.

Previous studies have found that following the introduction of the Public health act in Norway, municipalities have introduced new working methods and professionals and implemented organizational changes on both micro and macro levels [13, 14]. This includes appointment of public health coordinators [15] and a change towards increased understanding and adoption of the new, comprehensive public health policy [16]. Municipalities that developed health overviews reported more often to prioritising fair distribution of social and economic resources among social groups in political decision-making [17]. However, a countrywide supervision in 2015 showed that many of the municipalities had not decided how they wanted the CMOs to be involved in the work [18]. Also internationally there are tensions related to the role of CMOs [19], but no study focusing on the CMOs role in making overview documents has been identified.

The aim of this study was to investigate how Chief Medical Officers experience their role in the municipalities work with making the public health overview documents demanded by the Norwegian Public Health Act from 2012.

## Materials and methods

### Study design

This was a qualitative study with semi-structured focus group interviews with Chief Medical Officers, conducted in 2017. Focus groups were chosen as they foster discussions among the participants and are suitable to explore phenomena that concern common experiences, attitudes or views in a field where people interact [20]. The Consolidated criteria for Reporting Qualitative research checklist (COREQ) [21] was consulted for reporting the study.

### Participants and recruitment

The goal was to include CMOs from rural and urban areas in Norway, with variation in age, experience in public health, gender and size of the municipality they worked in. Eligible CMOs were recruited from the following three meetings. A yearly course arranged by the Chief County Medical Officer in a county in Central Norway, a compulsory course in the specialist program in community medicine held in Oslo and a workshop on new ways of organizing and managing GP practices in the western part of Norway. The identified participants were invited by e-mail which included information about the study.

### Data collection

**Fig. 1:**
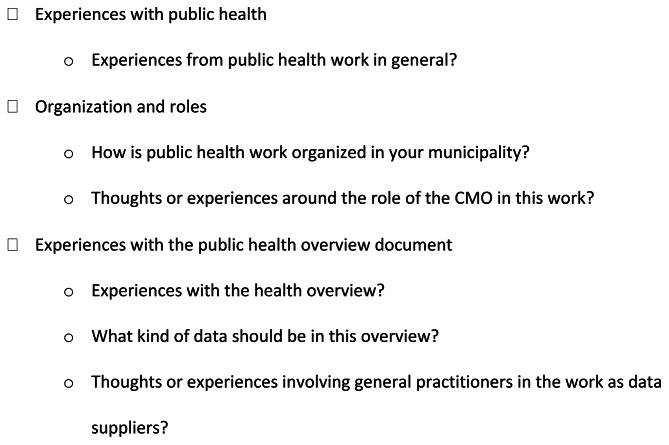
Interview guide

The data was collected in semi-structured focus group interviews, which lasted about 90 min and were audiotaped and transcribed verbatim. The participants were given verbal information about the study before the interviews started. The interview guide (Fig. 1) was developed based on relevant public documents related to public health. It was discussed with a group of 20 stakeholders interested in this topic, as well as discussions among the researchers. The main questions concerned experiences with public health work in general, and in particular the public health overview document.

### Analysis

Data were analyzed thematically, inspired by Braun and Clarke [22], in an iterative process. First, meanings and patterns were identified and discussed. Then meaning units related to the aim were coded and organized in themes. Seven initial main themes were identified (challenges related to the CMO-role in general, “yes the CMO is important”, intersectoral public health, challenges related to the CMO-role in public health, the role of GPs in public health, organizational conditions, public health vs. health care services). This process was repeated in an iterative process involving all authors and ended with three main themes (types of involvement, reasons for being involved or not, and the impact of the overview document).

## Results

A total of 21 CMOs participated in the three focus group interviews (Table 1). Twelve were specialists in community medicine/public health, and 11 worked fulltime as CMOs. Half of them were over 45 years old. The intended variation in geography and municipality size was reached. Exact data on years of experience from community medicine and public health were not systematically collected, but it varied from a few years to more than 30 years.

**Table 1:** Characteristics of the participants (*N* = 21)

Characteristic	Number
Age:	
- Age > 45 years old	11
- Age < 45 years old	10
Specialist in public health / community medicine:	
- Specialist	12
- Not specialist	9
Full time or part time position as CMO:	
- Full time position	11
- Part time position	10
Gender:	
- Male	14
- Female	7
Municipal size:	
- <5.000 inhabitants (small)	3
− 5.000 to 20.000 inhabitants (medium)	11
- >20.000 inhabitants (large)	7

### Types of involvement

Most of the informants expressed it as natural for them to participate in making the public health overview document, and that they had made a substantial contribution. A few had been leaders in the process, while others took a role as medical advisors. The main contributions were collecting, selecting and evaluating data, and making recommendations in the written report. Still almost none participated in all parts of the process, and some did not take part at all.

*I have given advice regarding structure, scope and working method. I wasn’t involved in the actual writing, but I know it was an extensive struggle to get data from the various sectors.*

It was said that having combined positions as GP made it easier to contribute to the collection of and ensuring the quality of the data from the health care sector. This included collecting data from local GPs including helping the GPs in extracting relevant data from the electronic health records. One senior CMO rejected using local data for this purpose because he did not find it trustworthy and would rather rely on national data. Some had interviewed GPs to get their input to the overview document.

*It was very rewarding* [collecting data from GPs]. *We had a series of meetings and the GPs received course credits, and it was just as good to discuss this as discussing other professional matters. So GPs are not hopeless in relation to thinking about public health work, I would say!*

The CMOs said that their role in the process was based on their knowledge about the municipality and the health care sector, and their relationships to other stakeholders in public health. One said that the CMO is a listening post to other sectors. Some emphasized their epidemiological training as important, giving them the ability to see relationships between data, do health impact assessments and understand the burden of disease in the local community. Also having skills in presenting knowledge, training in prioritizing and giving advice were mentioned as contributions.

*It is the competency in community medicine, to prioritize among many suggested proposals. An employee from the planning department may have a completely different understanding than the CMO. In a sense, it is in a group process the community medicine competency is relevant.*

### Reasons for being involved or not

The variation in what degree the CMOs had been involved in the process of making the public health overview documents, was mainly said to be due to organizational factors. Too small positions, a lack of tradition to involve the CMO in public health work, municipal directors with limited interests in public health, and limited access to other expertise locally were mentioned as barriers. One said he had deliberately chosen to let the public health coordinator take care of the process, so he could prioritize other tasks. Two CMOs in combined positions said it was hard to combine this kind of planning process with clinical work as GPs due to time constraint and difficulty in finding suitable meeting times. One talked about being involved as a medical alibi to legitimize the work, while another senior doctor felt being on the outside and left behind:

*From one perspective, it is good that the work involves more people, but my role has become more obscure. In some way, I have been somewhat degraded* […]. *The structure of public health work is no longer such that I am an explicit part of the leadership, which I think I should have been, even if it shall be distributed. And not just someone they ask when it suits them.*

Those who expressed being most involved described the process as rewarding. The work with the overview document was said to breathe new life into public health work, and giving the CMOs knowledge they would have missed without this process. The collaboration with the public health coordinators was especially mentioned as a valuable experience. One emphasized the importance of having office space in the same building as the public health coordinator. The intersectoral process was said to give good discussions with employees from other sectors, and through this work they saw that they gave politicians a good basis for making important decisions for the health of the population. Also, being organized at a cross-sectoral level with access to other stakeholders involved in public health, with enough time to interact with them, was seen as an advantage.

*It is important to be there and remind them of this* [the public health perspective] *when the decisions are made, and it is time-consuming, because then you have to take part in the planning processes and be involved early enough.*

### Impact of the health overview

There were different experiences as to the result of the process and how the overview document had been used after completion. One CMO said that she and her CMO colleague had created a web-based version of the overview document on the municipality’s website, with navigation features to make it more accessible. Several informants had witnessed how the process of making the overview document made employees in other sectors connect to public health in a new way. One had been included in intersectoral working groups where they used the overview document as a foundation for further municipal planning.

*When the planning program is approved by the politicians, we see many signs of the idea behind. We can now see the results from the work done by the public health coordinator and the CMO, having worked to collect this data that we have collected in a health overview, and that it will be used in the future.*

Still, several informants said that they did not see much use or impact of the public health overview document. Reasons given were that the politicians did not care, the plan was neglected and put in a drawer and the recommendations in the overview document were not prioritized in the municipality`s budget. The perspectives of the politicians were also commented. One CMO referred to politicians becoming very focused on measures concerning somatic health, asking “do we have more or less cancer than our neighbors?”. Others commented that they had a hard time making the politicians realize that effects of interventions in public health take time.

*But as it turned out, as soon as there was a cost connected to it, it went down to zero and nothing was granted for follow up. The plan was adopted, but interventions in the plan are completely dead. I haven´t bothered to get involved further since it got so negative from the administration and the politicians.*

## Discussion

The Chief Medical Officers said they were involved in various roles and to varying degrees in making public health overview documents. This was related to the size of their position as CMOs and different traditions in the municipalities to involve CMOs. How the municipalities used the overview document afterwards also varied.

### Study strength and limitations

Although no data were recorded about those invited who did not volunteer to participate, the characteristic of the participants indicates that the sought after variation was achieved. There was good variation in experiences, e.g. positive and negative attitudes towards public health work. Furthermore, the main topics were identifiable in all the interviews, which indicates that more interviews would not be likely to change the findings. Some of the informants were known by the authors’ networks and some knew each other. However, in the transcripts it was not found that the participants tried to please others, rather there were lively discussions about different experiences and viewpoints. This also shows that the use of focus groups was a good choice. All the authors were involved in the inductive analysis which ensured that they contributed with different perspectives and increased the likelihood of identifying the variations in the data. The data were collected before the Covid-19 pandemic, and it may be that interviews conducted after the pandemic would have given some different experiences. However, we assume that this would mainly concern infectious disease control, and that the experiences related to public health work would be similar to the situation in 2017.

### Health in all policy

The intention behind the public health overview documents and part of the public health act in Norway, is to formalize the implementation of health in all policies (HiAP) [13, 23]. None of the participants expressed any objection to this approach. On the contrary, the CMOs mainly expressed positive attitudes to the process of making public health overviews regardless of their own involvement. This is in accordance with an evaluation report that found that making overview documents contributed to a strengthened anchoring of public health in the municipal planning system and an increase in the use of resources for the organization and planning of public health work [24].

Some of the CMOs expressed an appreciation of and motivation for cross-sectoral work, but overall, there were few who talked about having a leading role in working with the overview. This is in line with previous evaluation of actions that is termed ‘intersectoral action for health’ (ISA), which concerns how the health sector works with other governmental and non-state sectors to improve health and well-being [25]. A Danish document analysis showed that the ideas that ISA builds on, has proven difficult to implement, and that the guidelines for municipal ISA were vague and constructed with buzzwords [26].

Furthermore, Health in all policy in Norway is nationally decided upon, but not fully recognized locally [27]. There are reports pointing out that Norwegian municipalities are at different levels regarding building competence necessary to achieve the objectives of the Public Health Act [28]. A report from 2015 concluded that the work required more resources, time, expertise and capacity than the municipalities had at their disposal [29]. It was stated that especially small municipalities did not have enough resources to meet the expectations from the governments. This is a likely reason for some of the experiences of the CMOs about the role of both the planning and use of the overview document.

### The role of the CMOs

There was a large variation of how involved the CMOs were in making of the public health overview documents, from hardly taking part, to having a central role. There were also comments about the changing role of the CMOs in the municipalities over time. One possible explanation is the increased focus on implementing inter-sectoral working groups and increasing numbers of public health coordinators. Such activities are used to coordinate and implement public health policies and measures in the municipalities, and are considered important when building capacity to reduce health inequities [30].

A major contribution from the CMOs that participated in the work, was the contact with GPs, both through helping to collect data from electronic health record and to interview GPs. This could in part be explained by the opportunities that come with CMOs working in combined positions [31], i.e. as both CMOs and GPs. Although CMOs have different attitudes towards their responsibility to go beyond clinical medicine to help even out social inequality in health [32], this activity points to a wish among the CMOs to use knowledge from the clinical work in primary care as a contribution to the population health perspective.

## Conclusion

Chief Medical Officers have experienced large variation in their involvement in making public health overview documents in Norwegian municipalities. More research is needed to understand if this has any consequences for the quality of public health work in the municipalities and whether it is a sign of a changing role of the Chief Medical Officers.

## Data Availability

The datasets generated and analysed during the current study are not publicly available due to data regulations but are available from the corresponding author on reasonable request.
